# Determining the Effect of Varied Proportions of Cohort Administered Tulathromycin at Arrival on Nasopharyngeal Microbiota and Performance Characteristics in Yearling Steers in the First 56 Days on Feed

**DOI:** 10.3390/microorganisms12122512

**Published:** 2024-12-05

**Authors:** Blaine T. Johnson, Brad J. White, Raghavendra G. Amachawadi, Michael D. Kleinhenz, Jaymelynn K. Farney, Teresa D. Shippy, Robert L. Larson

**Affiliations:** 1 School of Veterinary Medicine, Texas Tech University, Amarillo, TX 79106, USA; blaine.johnson@ttu.edu; 2 Beef Cattle Institute, College of Veterinary Medicine, Kansas State University, Manhattan, KS 66506, USA; rlarson@vet.ksu.edu; 3 College of Veterinary Medicine & Biomedical Sciences, Texas A&M University, VERO, Canyon, TX 79015, USA; mkleinhenz@tamu.edu; 4 Southeast Area Research and Extension Center, Department of Animal Sciences and Industry, Kansas State University, Parsons, KS 67357, USA; jkj@ksu.edu; 5 K-INBRE Data Science Center, Division of Biology, Kansas State University, Manhattan, KS 66506, USA; tshippy@ksu.edu

**Keywords:** bovine respiratory disease, tulathromycin, growth performance, nasal microbiota

## Abstract

Metaphylaxis or treating the entire population of cattle at arrival with an antimicrobial has been studied extensively in the cattle industry; however, little information is available on the impacts of treating only a proportion of the population with antimicrobials at arrival. The study objective was to determine potential associations between the proportion of animals in a pen treated with antimicrobial therapy with pen performance and nasopharyngeal microbiome. Yearling steers (n = 160) were randomly allocated to study pens (n = 40) and pens were systematically randomized to one of two antimicrobial treatments (META: all four head received tulathromycin; MIXED: two of four head randomly selected to receive tulathromycin). The study was conducted in conjunction with an essential oil feeding trial. Deep nasal pharyngeal (DNP) swabs were collected from every steer at Days 0, 14, 28, and 56. All DNP swabs were individually cultured for *Pasteurella multocida* and *Mannheimia haemolytica*. Samples of DNA were extracted from DNP swabs, pooled by pen, and analyzed by metagenomic shotgun sequencing to compare nasopharyngeal microbiome composition and quantity of resistance genes between test groups. Neither antimicrobial nor essential oil treatment groups had any significant associations with performance or DNP microbiome. Sampling day was significantly associated with alpha and beta diversity at the species level. Shannon’s diversity and Inverse Simpson diversity were significantly lower on Day 14 versus both Day 0 and Day 56. These data indicated a shift in microbial populations across study days; however, the microbiome diversity and relative abundance were not significantly different between antimicrobial treatment groups.

## 1. Introduction

Bovine respiratory disease (BRD) remains the costliest disease in the feedyard industry, causing substantial production and economic losses [1]. Numerous management strategies have been established to mitigate the effects of BRD on cattle production. Antimicrobial therapy of all cattle in a cohort at arrival (metaphylaxis) has been demonstrated as an effective method for reducing BRD total morbidity and subsequent mortality when administered to cattle deemed at high risk for BRD at arrival [2,3,4]. Selecting which groups of cattle need antimicrobial treatment at arrival based on information known at the time of administration is challenging [5]. Antimicrobials play a critical role in managing BRD in calves, but their use must be carefully weighed against the potential risks of promoting antimicrobial resistance [6]. However, the long-term reliance on metaphylaxis raises concerns regarding its impact on antimicrobial resistance (AMR). The widespread use of antimicrobials in feedlot settings can contribute to the selection and dissemination of resistant bacterial populations, posing challenges for both animal and public health [7]. Resistance genes can persist in microbial communities, potentially limiting future treatment options and exacerbating the AMR crisis [8,9]. Antimicrobial stewardship and judicious use of antimicrobials are crucial for preserving the effectiveness of these drugs in veterinary medicine. Public perception and antimicrobial stewardship are ongoing concerns for producers and veterinarians. One of the biggest obstacles of dealing with BRD is properly identifying diseased cattle [10]. Groups of cattle that are considered “high-risk” for BRD are often metaphylatically treated with an antimicrobial at arrival because the group is already clinically sick or has a high probability of being sick soon after arrival [11]. The inability to distinguish cattle with clinical or subclinical BRD from healthy animals results in treating the entire group with antimicrobials [2,3,12]. Most metaphylaxis research has required treating the entire cohort; however, recent information suggests targeted metaphylaxis of a portion of the pen based on physiologic parameters may have some benefits compared to no treatment [13,14]. This approach shows promise but involves additional procedures at initial cattle processing. Hanratty et al. (2023) illustrated randomly providing metaphylaxis at arrival at 0%, 33%, 66%, or 100% may have some benefit depending on the proportion of animals within the group treated [15]. These studies have evaluated potential impacts on health (morbidity and mortality) and performance metrics; however, the potential impacts of targeted or proportional metaphylaxis on the microbiome have not been evaluated. The advent of next-generation sequencing has allowed for a better understanding of the microbiota of the respiratory tract in cattle. Metaphylaxis has been shown to change the antimicrobial resistance pattern in stocker cattle, and research has demonstrated a difference in the respiratory tract microbiota of healthy vs. clinical BRD cattle [16,17,18]. There is evidence the nasopharyngeal population is highly correlated to the lower respiratory tract population in cattle [16]. Potential impacts of applying an antimicrobial to all or a proportion of the population should be better understood to fully evaluate the implications of the metaphylaxis decision relative to potential benefits. The primary study objective was to determine and characterize the nasopharyngeal bacterial populations within the first 56 days on feed based on treating all cattle or half the cattle within a pen with tulathromycin. The secondary objective was to determine potential performance differences associated with the antimicrobial treatment group within the first 56 days on feed. Additionally, this study was conducted simultaneously with the evaluation of the effects of essential oils on both nasal microbial populations and performance metrics to determine if adding essential oils to diets changes the nasal microbiome or performance characteristics.

## 2. Materials and Methods

All Experimental Procedures Were Approved by the Kansas State University Institutional Animal Care and Use Committee (Protocol No. 4566).

### 2.1. Animals and Facility

Yearling steers (n = 174) of angus-based genetics were purchased through auction markets in southeast Kansas in the week of 12 July through 16 July 2021. The cattle were trucked less than 50 miles to the Kansas State University Southeast research station in Mound Valley, KS, USA. Upon arrival, the cattle were rested for 48 h prior to processing (d 0). The cattle received long-stem hay and ad libitum access to fresh water. Prior to processing, the cattle were comingled into one group. Then, the cattle were processed, receiving vaccination for infectious bovine rhinotracheitis (IBR), bovine respiratory syncytial virus (BRSV), bovine viral diarrhea virus BVD (type 1 and type 2), parainfluenza-3 (PI3), and *Mannheimia haemolytica* toxoid (Pyramid 5 + Presponse^®^SQ—Boehringer Ingelheim), an oral broad-spectrum dewormer (Valbazen^®^-Zoetis) dosed at 4 mL/100 lbs (4 mL/45.45 kg), a radio frequency identification tag (Allflex^®^), a visual identification ear tag in the left ear, and a 150 mg trenbolone acetate and 21 mg of estradiol benzoate plus porous polymer film coating steroid growth promotion implant (Synovex One-Feedyard^®^, Zoetis) in the caudal subcutaneous portion, in the middle one-third of the cattle’s right ear. All injections were performed as per Beef Quality Assurance guidelines. Housing consisted of an open lot, dirt-floor pens, with a concrete bunk and apron. Pens were approximately 10.4 m × 30.5 m. Cattle had ad libitum access to shared automatic waters for the duration of the study period. Cattle were fed a common transition and finishing diet that met or exceeded Beef NRC guidelines for growth. The amount of feed delivered was monitored and adjusted every morning by feedyard personnel trained to make intake adjustments based on the cattle’s needs and consumption patterns. Feed intakes were optimized so that the bunks were completely empty of feed shortly after the next feeding. Cattle were fed twice a day for the duration of the feeding period.

### 2.2. Randomization and Treatment Administration

Out of 174 yearling steers purchased, the first 160 steers through the chute meeting the minimum weight requirement (340 kg) were utilized for the current study. Pen was used as the experimental unit and steer as the observational unit of this experiment. Pens were systematically ordered alternately by treatment group (META or MIXED; equating to 20 pens per treatment group; n = 20). Pens were then blocked into groups of 5, and the order within each block was randomized for the study’s essential oil diet treatment (0, 0.5×, 1×, 2×, and 4× levels, respectively). This placed 4 steers per pen, 8 pens per essential oil treatment, and 20 pens per antibiotic treatment group.

On processing day (d 0), steers were systematically randomized by chute order to pens to ensure equal distribution of potential contact between antibiotic treatment groups (4 steers per pen). In addition, cattle were further randomized within the META treatment group prior to the initiation of the study, d0, to receive the antibiotic treatment. In the META group, all cattle within pen (n = 4) were administered Tulathromycin at 2.5 mg/kg (Macrosyn, Bimeda^®^) at d 0. In the MIXED treatment group, half of the cattle (n = 2 out of 4) were administered Tulathromycin at 2.5 mg/kg at d 0. Randomization occurred using the randomizeR package, version 4.2.1 [19] in the R programming language [20].

### 2.3. Sample Collection

#### Deep Nasopharyngeal Swabs

Nasopharyngeal swabs were collected on d 0, d 14, d 28, and d 56 of the current study by a trained veterinary professional. All cattle were swabbed with a 59 cm guarded polyester swab (Double-Guarded Mare Uterine Culture Swabs, Jorgensen Laboratories Inc.) to collect deep nasopharyngeal samples. Nare debris were removed and swabs were inserted to a depth approximately at the location of the medial canthus in either the right or left nostril of the steer and gently used to scrub the mucosa for sample collection. Swabs were then placed into labeled 14 mL round-bottom falcon tubes with approximately 1.5–2 mL of Amies transport media [21], placed on ice, and transported three and half hours to Kansas State University (Manhattan, KS, USA) for processing and storage. Individual tubes were grouped by pen, placed in appropriate holding boxes, and stored at −80 °C until processing for culture and genomic analysis. Authors elected to only analyze nasopharyngeal samples from d 0, 14, and 56 for culture and genomic sequencing due to financial restrictions.

### 2.4. Bacterial Isolation and Identification of Pasteurella multocida and Mannheimia haemolytica

From the individual nasal swab samples, the isolation of *Pasteurella* spp. and *M. haemolytica* were carried out as per the procedure outlined in [22,23], respectively. Two isolates of each nasal pathogens exhibiting a typical colony morphology were subjected to catalase and oxidase biochemical tests. Genus and species confirmation of *P. multocida* and *M. hemolytica* was achieved by PCR [24]. The confirmed isolates (two isolates of each species per sample) were stored in Cryocare-protect^®^ beads (Key Scientific Products, Stamford, TX, USA) at −80 °C for future use.

### 2.5. Antimicrobial Susceptibility Determinations of Isolated Bacteria

Minimum inhibitory concentrations (MICs) were determined by the broth microdilution method as per CLSI guidelines [25], using the Sensititre^®^ automated antimicrobial system (Trek Diagnostics Systems, Cleveland, OH, USA). The commercially available National Antimicrobial Resistance Monitoring System (NARMS) Bovine Tulathromycin MIC format Sensititre^®^ (BOPO-7F) panel plate (ThermoFisher Scientific, Lenexa, KS, USA) was used with the aid of the Sensititre^®^ automated inoculation delivery system (Trek Diagnostics Systems, Cleveland, OH, USA). Appropriate ATCC (American Type Culture Collection, Manassas, VA, USA) quality control strains, *Escherichia coli* ATCC 25922, and *Enterococcus faecalis* ATCC 29212 were used as reference standards for susceptibility testing. The MIC for each isolate were recorded and classified as resistant, intermediate, or sensitive based on the CLSI guidelines.

### 2.6. DNA Extraction from Nasal Samples

Total DNA was extracted from each swab sample and grouped to a pen level using the “PowerSoil^®^ DNA isolation kit” (MO BIO Laboratories; Carlsbad, CA, USA) according to the manufacturer’s protocol. The isolated DNA was stored at −20 °C until used for metagenome shotgun sequencing.

### 2.7. Library Preparation and Sequencing

Genomic DNA samples were quality-checked with the HS dsDNA Qubit assay for quantification, and Agilent TapeStation gel analysis was performed for gDNA quality. Sequencing libraries were constructed using 100–500 ng of gDNA using the Illumina DNA Prep sequencing library kit (Illumina, San Diego, CA, USA). The sequencing library construction includes tagmentation of the gDNA using a bead-based transposome complex to simultaneously fragment and tag the DNA with adapter sequences. Following tagmentation, unique dual-index adapters are added in a PCR amplification step to the ends of the DNA fragments. The constructed sequencing libraries were quantified and validated with Qubit and TapeStation assays. After pooling the sequencing library preps together equally by ng amount, the nM concentration of the pool was verified with an Illumina Library Quant qPCR assay (Roche, Indianapolis, IN, USA). An Illumina NextSeq2000 system at the University of Kansas’s Genome Sequencing Core was used to generate ~1.1 billion paired-end, 150-base sequence reads from the multiplexed libraries. Base calling was carried out by the instrument’s Real-Time Analysis (RTA) software (version 3). The base call files (bcl files) were demultiplexed and converted to compressed FASTQ files using the on-board DRAGEN BCL Convert software (version 4.2.7).

### 2.8. Statistical Analysis

#### 2.8.1. Feedyard Performance Analysis

Feedyard performance was assessed for the first 56 days on feed. Linear models were created using the lm function in the R programming language. Continuous outcomes such as average daily gain and gain to feed were set as dependent variables, and antimicrobial treatment, diet, and their respective interaction were set as independent variables. The interaction of antimicrobial by diet (essential oil treatment) was not significant (*p* > 0.05) and thus excluded from the remainder of the analysis.

#### 2.8.2. Culture and Susceptibility Analysis

A mixed-effects logit model was performed for calculating the odds ratio of culturing *P. multocida* based on treatment group and timepoint. The interaction between treatment and timepoint was tested and found not significant.

#### 2.8.3. Bioinformatics Analysis

The reads were analyzed using the AMR++ 2.0 pipeline [26] run on Beocat, the Kansas State University High Performance Computing Cluster. As part of this pipeline, raw reads were trimmed for the removal of adapters and low-quality bases using Trimmomatic (version 0.39) [27]. Less than 1% of the reads were removed because of low quality. Burrows–Wheeler Alignment (bwa) [28] was used to align high-quality trimmed reads to the host Bos taurus genome [the ARS-UCD1.2 female reference genome (GCF_002263795.2_ARS-UCD1.3_genomic.fna.gz)] plus the Y chromosome (*Bos_taurus*_Y_CM001061.2. fasta). BEDTools, version 2.31.0 [29] was used to remove reads aligning to the host genome from further analysis. All samples had over 99.8% host (bovine) reads, so less than 0.2% of the trimmed reads were nonhost reads. The nonhost reads were taxonomically classified using Kraken 2 [30] with the Standard Kraken database (20220607 version). To identify reads matching antimicrobial resistance (AMR) genes and screen for single-nucleotide polymorphisms (SNPs) in those reads, we used ARIBA v2.14.6 [31] with reference sequences from the CARD database, version 3.2.4 [32] and AMR++ v3.06 with MEGARes v3.0 [33]. Taxonomic data from Kraken 2, consisting of read counts for each sample and a metadata file including raw read counts, nonhost read counts, and group information for each sample, were imported into R [20] for statistical analysis. Since the read counts were not normally distributed, we used non-parametric methods to test for significant differences in raw and nonhost read counts between the antimicrobial treatment group, essential oil treatment group, and sampling day. For factors with two groups, we used the Wilcoxon test. For those factors with three or more groups, we used the Kruskal–Wallis test and performed post hoc pairwise testing using Dunn’s test with Holm–Bonferroni multiple testing correction if the Kruskal–Wallis test indicated a significant difference. Before normalization and further statistical analysis, the read count table from Kraken 2 was filtered to keep only bacteria with at least one count in at least 5% of samples. The remaining counts were normalized by the Cumulative Sum Scaling method using the *cumNorm* function of the *metagenomeSeq* package [34]. These normalized counts were aggregated to the species, genus, and phylum levels with the *tax_glom* function of *phyloseq* [35]. All bovine nasal metagenomics sequence data from this study have been deposited under NCBI BioProject ID PRJNA1049020.

### 2.9. Relative Abundance

Normalized, aggregated counts were used to calculate relative taxa abundance at the species, genus, and phylum levels. For species and genus, taxa with a relative abundance less than 0.005 were grouped together to simplify analysis. Plots of relative abundance were created using the *geom_bar* function of *ggplot2* version 3.5.1 [36].

### 2.10. Alpha Diversity

Alpha diversity was calculated at the species, genus, and phylum levels using the *estimate_richness* function of *phyloseq* version 3.20. The alpha diversity index distributions did not meet assumptions of normality, particularly for residuals, so we used non-parametric tests to check for differences between groups. Analysis was performed as described for sequencing read counts in Section 2.8.3.

### 2.11. Beta Diversity

To visualize the relationships between samples based on beta diversity, non-metric multidimensional scaling (NMDS) plots were created in the *vegan* package version 2.6-8 [37] using the *ordinate* function (version 1.16.2) on the Bray–Curtis distance. NMDS is based on rank order of differences and can use specialized distance measures such as Bray–Curtis dissimilarity. Ellipses showing 95% confidence intervals for a multivariate t-distribution were added using the *stat_ellipse* (version 1.0.1) function of *ggplot2* version 3.5.1.

To test for significant differences between the centroids of groups, we used PERMANOVA [38] via the *adonis* function of *vegan* (version 2.6-4), since PERMANOVA makes no assumption of normality. Post hoc pairwise PERMANOVA testing was performed using the *pairwise.adonis* function (version 0.4) when the global PERMANOVA test for a group showed a significant difference. Multiple testing correction was performed with the Holm–Bonferroni method, which provides moderately stringent correction.

### 2.12. Differential Abundance Testing

To test for differential abundance of individual species between groups of samples, we used the *ancombc2* function of the *ANCOM-BC* package version 3.20 [39,40] since it is designed for use with compositional data, corrects for sampling and sequencing biases common in microbiome data, and can compare multiple groups. The Holm–Bonferroni method [41] was used for multiple testing correction to obtain a q value. Plots of individual species abundance were created using the *featurePlot* function of R (version 2.3.4).

## 3. Results

A total of 160 cattle were enrolled in the current study in 40 pens. A total of six cattle succumbed to a heat stress event that hit the region of SE Kansas on 28 July and 29 July 2021. Five of the cattle were from pens in the MIXED treatment group and one was from the META treatment group. Those pens (n = 6) were removed from the final analysis. The subsequent performance data (ADG, Feed:Gain) did not differ by antimicrobial treatment group (META vs. MIXED), essential oil treatment, or the antimicrobial group by essential oil treatment interaction (*p* > 0.05) for all periods of the study (Table 1).

Nasopharyngeal swabs bacterial cultures identified 49 samples of *Pasteurella multocida* and 2 samples of *Mannheimia haemolytica* over the three time periods (0, 14, and 56 days on feed) and confirmed by PCR (Table 2).

**Table 2:** Confirmed PCR bacterial isolates from cultures of deep nasopharyngeal swabs across timepoints and treatment groups of Tulathromycin administration in yearling steers.

*Pasteurella multocida*				
		MIXED ^a^	META ^b^	Total
	Period			
	D0	1	1	2
	D14	9	3	12
	D56	16	19	35
Total		26	23	49
*Mannheamia haemolytica*				
		MIXED	META	Total
	Period			
	D0	0	0	0
	D14	0	1	1
	D56	0	1	1
Total		0	2	2

^a^ Half of the cattle within each pen (n = 2) received Tulathromycin at d 0 per labeled dosage. ^b^ All cattle within each pen (n = 4) received Tulathromycin at d 0 per labeled dose. The interaction between timepoint and antimicrobial treatment was not significant. The probability of culturing *P. multocida* or *M. haemolytica* did not differ by antimicrobial treatment group; however, timepoint of cultures was found to have a significant effect on the probability of culturing *P. multocida* (*p* < 0.01, Table 3).

At Days 0, 14, and 56, *Pasteurella multocida* isolates were cultured and analyzed for antimicrobial resistance in two treatment groups: “MIXED” (50% of the pen received Tulathromycin) and “META” (100% of the pen received Tulathromycin). At Day 0, no *P. multocida* isolates were detected in both groups. By Day 14, the “MIXED” group had three isolates, showing resistance to Penicillin (PEN) and Spectinomycin (SPEC), while the “META” group had nine isolates, with resistance to Tulathromycin (TULA) in two isolates. At Day 56, the “MIXED” group had 19 isolates with resistance to Oxytetracycline (OXY), Penicillin (PEN), and Spectinomycin (SPEC), and 1 isolate resistant to Tulathromycin (TULA). The “META” group had 16 isolates, with resistance to Tulathromycin (TULA) in 4 isolates. These results highlight varying resistance patterns between the treatment groups, with increased resistance to Tulathromycin over time in the “META” group (Table 4).

Notably, *M. haemolytica* isolates showed intermediate resistance to Spectinomycin at Day 56, with an MIC value of 32 µg/mL, and to Tulathromycin, with an MIC value of 8 µg/mL. These findings indicate stable susceptibility to most antimicrobials over the study period, with some variation in resistance patterns observed, particularly to Spectinomycin and Tulathromycin at Day 56 (Table 5). One *Mannheimia haemolytica* isolate from Day 14 was susceptible to all the tested antimicrobials.

**Figure 1:**
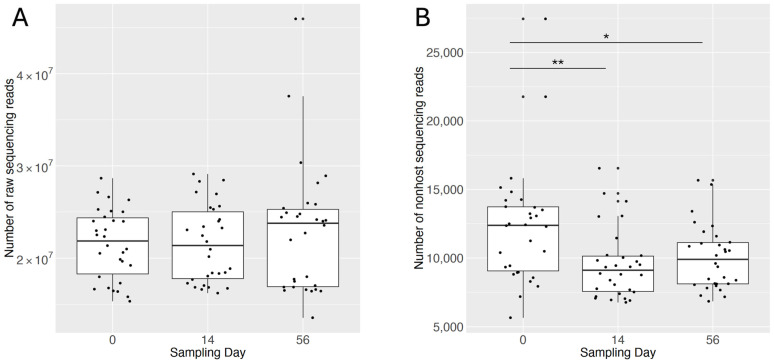
Boxplots illustrating the distribution of read counts by sampling day for (**A**) raw reads and (**B**) nonhost reads. Each sample is represented by a dot plotted with horizontal jitter to minimize overlap. Post hoc pairwise comparisons with significant adjusted P values are indicated by horizontal lines with asterisks (* = <0.05 and ** = <0.01).

The total genomic DNA from 90 bovine nasal swabs was sequenced to obtain 1,980,732,256 reads (990,366,128 paired reads). Reads matching the bovine genome were removed from the analysis, leaving 954,668 nonhost reads. Nonhost reads per sample ranged from 5660 to 27,452, with an average of 10,607 nonhost reads per sample. Using multiple univariate linear models, sampling day, antimicrobial treatment group, and essential oil treatment group were tested for any significant associations with total and nonhost read counts. Sampling day was associated with nonhost read count, but not total read count. Post hoc pairwise comparisons indicated nonhost reads were significantly higher on Day 0 than on Days 14 and 56, but not significantly different between Day 14 and Day 56 (Figure 1).

**Figure 2:**
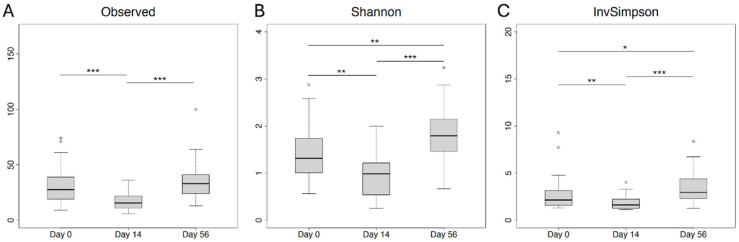
Alpha diversity boxplots. Comparison of (**A**) observed, (**B**) Shannon, and (**C**) Inverse Simpson alpha diversity metrics among samples from Days 0, 14, and 56. Post hoc pairwise comparisons with significant adjusted *p* values are indicated by horizontal lines with asterisks (* = <0.05, ** = <0.01, and *** = <0.001).

No significant differences were identified in nonhost read counts between the antimicrobial treatment groups or essential oil treatment groups. Nonhost reads were taxonomically classified using Kraken 2, with an average of 9.73% of non-bovine reads being classified. Before performing additional analysis, all non-bacterial and rare taxa (those with counts in fewer than 5% of the samples) were removed. After normalization of counts between samples, taxa were aggregated to allow analysis at the species, genus, and phylum levels. These aggregated taxa list included 224 species, 136 genera, and 13 phyla, respectively. The effect of sampling day, antimicrobial treatment group, and essential oil treatment group on alpha and beta diversity at the species level were assessed via univariate analysis. Only sampling day was significantly associated with each outcome (Figure 2). Species richness (observed), Shannon’s diversity, and Inverse Simpson diversity were all significantly lower on Day 14 than on Day 0 or Day 56. Day 0 and Day 56 were also significantly different for both Shannon’s and Inverse Simpson diversity, with Day 56 having a higher diversity index in both cases.

**Figure 3:**
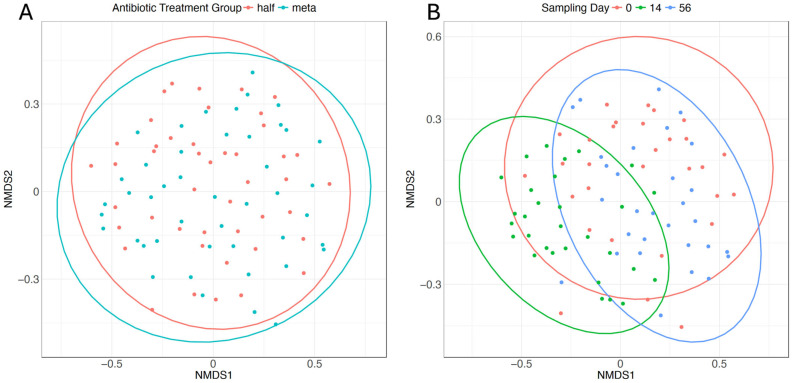
Species beta diversity. NMDS plots of species beta diversity by (**A**) sampling day, or (**B**) antimicrobial treatment. Ellipses denote 95% confidence intervals. Pairwise PERMANOVA analysis indicates significant differences in beta diversity for all pairwise comparisons of sampling day: Day 0–Day 14, R^2^ = 0.075, adjusted *p* = 0.003; Day 0–Day 56, R^2^ = 0.052, adjusted *p* = 0.003; Day 14–Day 56, R^2^ = 0.159, adjusted *p* = 0.003. There is no significant difference between antibiotic treatment groups (R^2^ = 0.017, *p* = 0.133).

Species beta diversity was significantly different for all pairwise adonis comparisons between sampling days. The non-metric multidimensional scaling (NMDS) ordination (Figure 3) plot shows the clustering of samples by day, with Day 14 forming the most distinct cluster (Figure 3A).

**Figure 4:**
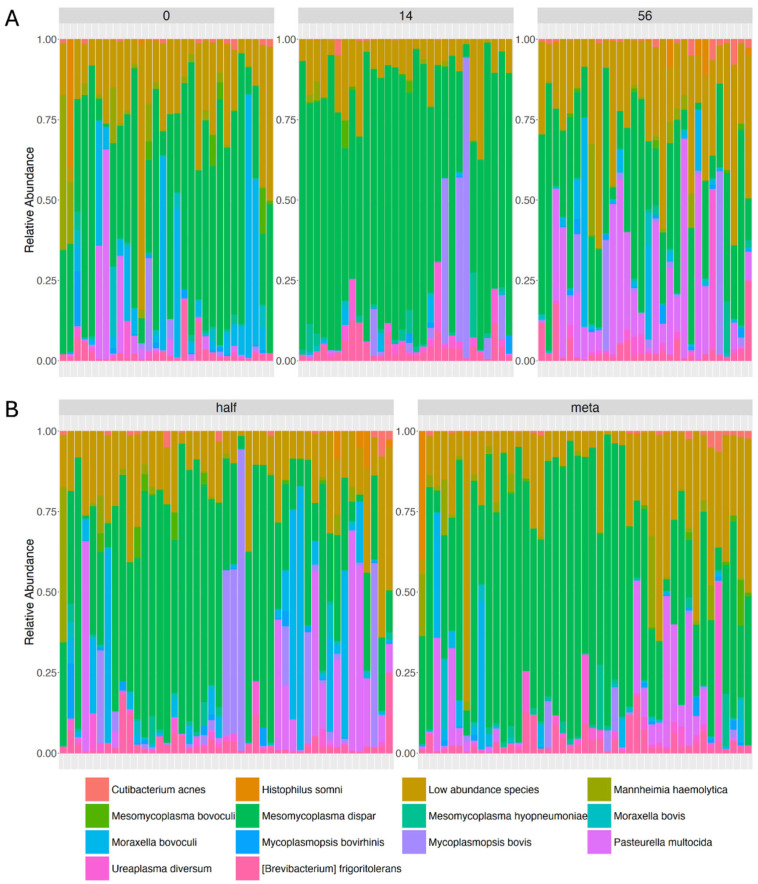
Species relative abundance. Relative abundance of species in samples grouped by (**A**) sampling day or (**B**) antimicrobial treatment. Panels are labeled by group: Day 0, 14, and 56 in A and half and meta in B. Each vertical bar represents one sample. Species are color-coded according to the accompanying key, with species with relative abundance under 0.005 grouped as “low-abundance species”.

To gain further insight into differences in composition between samples, we compared the relative abundance of species in samples grouped by sampling day (Figure 4A) or antimicrobial treatment (Figure 4B). Species with less than 0.5% overall relative abundance were grouped in a single “low-abundance species” category. Differential abundance of individual species was also tested using ANCOM-BC2 version 3.20 [39,40]. All species present in more than 5% of samples were considered in this analysis. No species were significantly different between the antimicrobial treatment groups or essential oil treatment groups.

**Figure 5:**
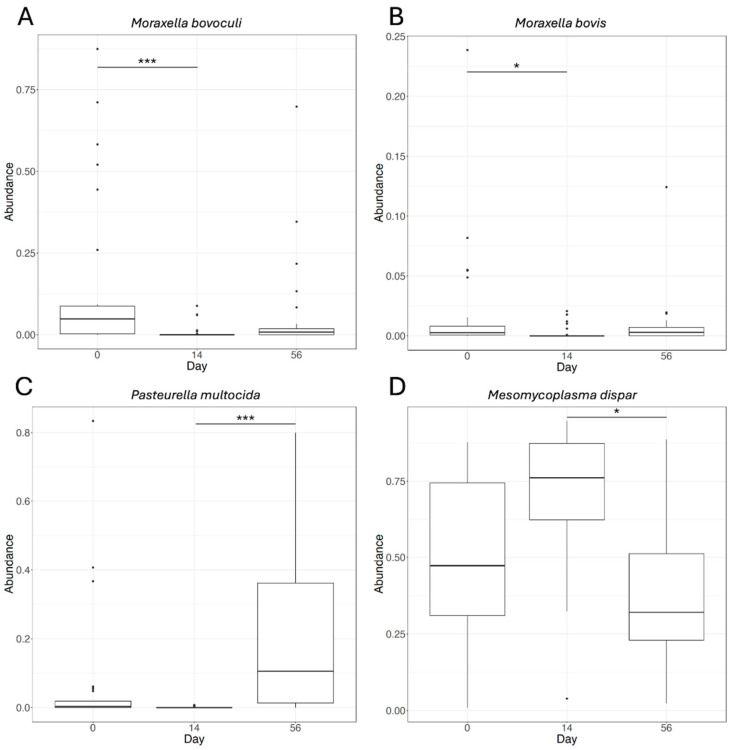
Differential species abundance by day. Feature plots of relative abundance by sampling day for the four species with statistically significant differential abundance between sampling days (**A**) *Moraxella bovoculi*, (**B**) *Moraxella bovis*, (**C**) *Pasteurella multocida*, and (**D**) *Mesomycoplasma dispar*. Significant differences in abundance (after Holm–Bonferroni correction) are indicated by horizontal lines with asterisks (* = <0.05 and *** = <0.001).

Four species showed significant differential abundance in the pairwise comparison of sampling days (Figure 5). *Moraxella bovoculi* (Figure 5A) and *Moraxella bovis* (Figure 5B) showed significantly higher abundance on Day 0 than on Day 14. *Pasteurella multocida* (Figure 5C) and *Mesomycoplasma dispar* (Figure 5D) were significantly different between Day 14 and Day 56, but showed opposite effects. *P. multocida* was lower on Day 14 and higher on Day 56, while *M. dispar* was higher on Day 14 and lower on Day 56.

### AMR Genes

We used AMR++ and ARIBA to identify potential antimicrobial resistance (AMR) genes among the nonhost genes and to check for the presence of known variants in these genes. Only one sample had a confirmed variant known to be associated with AMR. AMR++ detected five reads in a sample from one cattle with a mutation in 23s rRNA known to confer resistance to macrolides.

## 4. Discussion

Antimicrobial drugs are important tools for maintaining human and animal health [42]. Previous research in humans suggests that viral regulation of microbial communities in the upper respiratory tract (URT) may influence the transition of certain members to pathogens and impact host immune responses [43]. Kang et al. (2023) [44] also highlights the importance of antimicrobials in human medicine and animal agriculture, emphasizing the need for reliable and quality standards to ensure efficacy and prevent adverse effects.

Consideration of the potential for the selection of antimicrobial resistance should be emphasized when considering antimicrobial selection in the treatment of BRD in calves [6]. Furthermore, antimicrobials remain a common and effective tool for the reduction of BRD in feedyard production. The purpose of this study was to evaluate impacts of varied proportions of the cohort treated metaphylactically with tulathromycin on pen performance and the nasal microbiome. An interaction between the two treatments administered in the trial (antimicrobial and essential oils) was not observed; therefore, all results are interpreted by treatment. The proportion of pens treated with antimicrobials did not impact performance, indicating that the effects of the essential oils may have influenced the overall outcomes independently of the antimicrobial treatment. The antimicrobial treatment was not associated with the nasal microbiome; however, several significant associations between measured parameters and trial day were identified. The lack of differences in the nasopharyngeal microbiome between antimicrobial treatment groups is an unexpected finding that requires further exploration. Several factors may explain this result, such as the inherent resilience of the nasopharyngeal microbiome, variations in antimicrobial effects across individuals, or external environmental influences. Expanding on these aspects could provide additional insights into this counterintuitive outcome.

Metaphylactic antimicrobial administration has proven benefits through decreasing morbidity and mortality. This trial consisted of heavier, older animals which would often be considered at lower risk for BRD and associated illness [45]. No morbidity was identified in this trial and the mortalities identified were the result of non-infectious disease. The lack of differences in ADG and feed performance identified between the two antimicrobial treatment groups could be due to the low baseline disease challenge and little potential advantage to a higher proportion of the pen treated with antimicrobials.

Bacterial cultures focused on identifying two key BRD pathogens: *Pasteurella multocida* and *Mannheimia haemolytica* at each time period. No differences were identified by antimicrobial treatment group; however, *Pasteurella multocida* was more likely to be cultured at Day 14 vs. Day 0, Day 56 vs. Day 0, and Day 56 vs. Day 14. These results indicate that in both antimicrobial treatment groups, the likelihood of culturing *Pasteurella multocida* was higher as time went on during the feeding period, which could be due to longer contact of animals between and within pens. *Mannheimia haemolytica* isolates from Day 14 (META) did not show significant resistance to any of the antimicrobials tested. However, *M. haemolytica* isolates from Day 56 (META) showed subtle development of resistance to the tested antimicrobials. Overall, these results highlight the persistence of susceptibility to most antimicrobials over time, although a slight increase in resistance was observed for Spectinomycin and Tulathromycin at Day 56. This shift may be related to the duration of Tulathromycin treatment, underscoring the need for further research to explore the long-term impact of antimicrobial use on resistance patterns in *Mannheimia heamolytica* isolates from cattle. The results indicate that both antimicrobial treatment strategies—MIXED and META—led to the emergence of antimicrobial resistance over time. While no *Pasteurella multocida* isolates were present at Day 0, resistance to key antibiotics such as Penicillin, Oxytetracycline, and Tulathromycin emerged by Day 14 and Day 56. Notably, the META group, which received Tulathromycin for the entire pen, showed higher resistance to this antimicrobial, suggesting potential selective pressure. The development of resistance to other antibiotics in the MIXED group, despite the limited use of Tulathromycin, highlights the complex dynamics of antimicrobial resistance and the need for careful management of antimicrobial use in livestock.

Total nonhost genomic reads were significantly lower on Day 14 and Day 56 compared to Day 0 although reads counts were not associated with the antimicrobial treatment group. Lower nonhost reads after antimicrobial administration has been reported in this study, and the cohort level nonhost reads did not differ if two out of four cattle were administered an antimicrobial compared to four out of four cattle. The lack of interaction between antimicrobial and essential oil treatments indicates that essential oils may have influenced the overall outcomes independently of antimicrobial treatment, as the proportion of pens treated with antimicrobials did not affect performance. This aligns with previous findings on essential oils and nasal microbiota, showing that a single intranasal dose of EO spray moderately modulated the nasopharyngeal microbiota and temporarily inhibited *Mannheimia* without affecting animal performance [46]. These findings differ slightly from alpha diversity measures which, while lower at Day 14 than Day 0, showed a return to levels on Day 56 that were not different from Day 0 when Inverse Simpson alpha diversity was evaluated. Both findings illustrate an impact of antimicrobial administration on the nasal microbiome similar to previously reported work; however, the lack of difference between antimicrobial treatment groups is interesting [16,21,47]. This could be due to the relatively small sample size determined by the number of pens, or this finding could be related to pen dynamics in relatively small pens, where the nasal microbiome might transfer between cattle in different treatment groups. Another possibility is that the close proximity in these settings facilitates microbiome sharing among cattle. These findings are further illustrated by the measures illustrating differences in species beta diversity among sampling days but not between antimicrobial treatment groups. Visual evaluation of relative diversity plots illustrated changes over time similar to alpha and beta diversity evaluations, but few differences among antimicrobial treatments. In aggregate, these findings may indicate that the cohort impact of antimicrobial administration may not be reliant on treating all individuals within a group.

Only one sample had a confirmed variant known to be associated with AMR; therefore, no further analysis was performed to evaluate potential associations with treatment or timing. Previous work has illustrated changes in antimicrobial resistance patterns following administration of metaphylaxis, but this finding was not replicated in the current study. Overall, it is important to understand the relationship between antimicrobial use practices, microbiome composition, and AMR in livestock production systems [48]. It underscores the need for comprehensive strategies to combat AMR, including prudent antimicrobial use, surveillance of AMR in both animals and humans, and continued research to elucidate the mechanisms underlying AMR emergence and dissemination in agricultural settings [48]. By addressing the complex interplay between antimicrobial use, microbial ecology, and AMR, stakeholders can work towards sustainable and responsible antimicrobial stewardship practices to safeguard both animal and human health [48].

Limitations of this work include small number of cattle (n = 4) per pen and several pens being removed from analysis due to the loss of individuals from a heat stress event. The small pen dynamics may differ from larger pens relative to potential impact on the nasal microbiome over time; however, these results did not show differences based on the proportion of antimicrobials administered at the cohort level (two out of four versus four out of four cattle). In addition, larger-grouped pens would also add observational units to flush out any (if any) pen variation compared the small sample size in pens and provide more robust estimates. Pens removed due to the heat loss event made the study an unbalanced study. This carries the potential for study bias and reduced the statistical power initially planned for the study. However, the authors believe that the imbalance in experimental units was appropriately handled through the analysis methods used for this study, and a priori power calculations resulted in adequate power for the study even in the face of lost experimental units. Furthermore, cattle used in this study would be considered relatively low-risk for BRD. Generalizations from these data should not be applied to cattle that do not face a similar risk of BRD.

## 5. Conclusions

In conclusion, this study evaluated the impact of varying proportions of tulathromycin treatment on nasopharyngeal microbiota and performance characteristics in yearling steers during the first 56 days on feed. No significant differences in performance metrics (e.g., ADG, feed intake) or the nasal microbiome were observed between the two treatment groups; however, significant temporal changes in bacterial populations, particularly *Pasteurella multocida*, were noted over the trial period. The study also observed changes in nonhost genomic reads and alpha diversity measures, particularly a reduction after tulathromycin administration, though no differences were detected between treatment groups. These findings suggest that antimicrobial effects on the microbiome may not be strictly dose-dependent at the cohort level. Importantly, no antimicrobial resistance (AMR) variants were identified, aligning with previous work suggesting limited short-term AMR development post-metaphylaxis in low-risk cattle. As a future direction, we plan to explore varied cohort compositions in our studies, including factors such as age, health status, and environmental exposure, to better understand their influence on outcomes. Additionally, we aim to investigate the impact of different antimicrobial dosages and treatment durations to refine our understanding of their effects on the nasopharyngeal microbiome and overall efficacy. These efforts will help contextualize our current findings and provide a foundation for more tailored and effective antimicrobial strategies in the future. Sustainable antimicrobial stewardship in livestock production is essential for mitigating AMR while maintaining animal and public health.

## Figures and Tables

**Table 1:** Weight and performance data from 56-day yearling steer data at the pen level evaluating potential differences by antimicrobial therapy treatment group (META, all cattle in the pen received tulathromycin; MIXED, 2 of 4 cattle in the pen received tulathromycin).

	MIXED	META	*p* Value
Pens	16	19	
Arrival weight, kg	412.88	413.25	
SE, kg	4.36	3.95	
Day 14 weight, kg	439.91	439.44	0.95
SE, kg	5.43	4.62	
Day 28 weight, kg	468.05	466.18	0.81
SE, kg	5.88	5.18	
Day 56 weight, kg	502.79	504.98	0.77
SE, kg	5.72	4.79	
ADG d 14, kg	2.38	1.85	0.32
SE, kg	0.54	0.18	
ADG d 28, kg	1.97	1.89	0.56
SE, kg	0.08	0.10	
ADG d 56, kg	1.24	1.39	0.35
SE, kg	0.09	0.12	

Six pens (6) from the original 40 were removed from analysis due to heat event deaths (Pens 8, 9, 12, 16, and 26), respectively. SE = standard error. ADG = Average Daily Gain.

**Table 3:** Post hoc comparison of PCR-positive nasopharyngeal cultures of *Pasteurella multocida* from yearling steers (n = 120 cattle) at two pen levels of Tulathromycin injections (half or all (META)) over three timepoints.

Comparison	Odds Ratio	Lower Confidence Limits	Upper Confidence Limits	*p*-Value
Day 0 vs. Day 14	0.15	0.034	0.935	0.04
Day 0 vs. Day 56	0.04	0.007	0.23	<0.01
Day 14 vs. Day 56	0.26	0.11	0.63	<0.01

No differences were reported by antimicrobial treatment group. Timepoints correspond to days on feed, with Day 0 being the treatment allocation day. A Tukey method of adjustment was applied for the adjustment of family error.

**Table 4:** Antimicrobial susceptibilities of Pasteurella multocida (n = 49) isolated from deep nasopharyngeal swabs of yearling steers treated with Tulathromycin at different dosages and timepoints.

DOF ^1^	N ^2^	TRT ^3^	AMP ^4^	CEFT ^4^	CHLR ^4^	DANO ^4^	ENRO ^4^	FLOR ^4^	OXY ^4^	PEN ^4^	SPEC ^4^	TULA ^4^
0	1	MIXED	0	0	0	0	0	0	0	0	0	0
14	3	MIXED	0	0	0	0	0	0	1	0	1	1
56	19	MIXED	0	0	0	0	0	0	4	0	9	1
0	1	META	0	0	0	0	0	0	0	0	0	0
14	9	META	1	0	0	1	1	0	2	0	2	1
56	16	META	0	0	0	0	0	0	4	0	7	1

^1^ DOF = days on feed. ^2^ N = number of cultured and PCR-confirmed *Pasteurella multocida* isolates. ^3^ TRT = antimicrobial treatment group; “MIXED” = 50% of pen randomly received Tulathromycin at processing per labeled directions; “META” = 100% of pen received Tulathromycin at processing. ^4^ AMP = Ampicillin; CEFT = Ceftiofur; CHLR = Chlortetracycline; DANO = Danofloxacin; ENRO = Enrofloxacin; FLOR = Florfenicol; OXY = Oxytetracycline; PEN = Penicillin; SPEC = Spectinomycin; TULA = Tulathromycin.

**Table 5:** Minimum inhibitory concentration (MIC) values for various antimicrobials against *Mannheimia haemolytica* isolates (n = 2) from yearling steers treated with Tulathromycin, assessed at Days 14 (META D14) and 56 (META D56). The MIC values are categorized into susceptible, intermediate, and resistant ranges for each antimicrobial tested.

Antimicrobials	MIC Susceptible	MIC Intermediate	MIC Resistant	ID 5 META D14	ID 103 META D56
Ampicillin	≤0.03	0.06–0.12	≥0.25	<0.25	<0.25
Ceftiofur	≤2	4	≥8	<0.25	<0.25
Chlortetracycline	≤2	4	≥8	<0.5	<0.5
Danofloxacin	≤0.25	0.5	≥1	<0.12	<0.12
Enrofloxacin	≤0.25	0.5–1	≥2	<0.12	<0.12
Florfenicol	≤2	4	≥8	1	0.5
Oxytetracycline	≤2	4	≥8	1	<0.5
Penicillin	≤0.25	0.5	≥1	0.5	1
Spectinomycin	≤32	64	≥128	32	32
Tilmicosin	≤8	16	≥32	8	8
Tulathromycin	≤16	32	≥64	8	8

## Data Availability

All bovine nasal metagenomics sequence data from this study have been deposited under NCBI BioProject ID PRJNA1049020.
